# Case Report: Co-Existence of BRCA2 and PALB2 Germline Mutations in Familial Prostate Cancer With Solitary Lung Metastasis

**DOI:** 10.3389/fonc.2020.564694

**Published:** 2020-10-26

**Authors:** Tang Tang, Lin-ang Wang, Peng Wang, Dali Tong, Gaolei Liu, Jun Zhang, Nan Dai, Yao Zhang, Gang Yuan, Kyla Geary, Dianzheng Zhang, Qiuli Liu, Jun Jiang

**Affiliations:** ^1^ Department of Urology, Daping Hospital, Army Medical University, Chongqing, China; ^2^ Cancer Center, Daping Hospital, Army Medical University, Chongqing, China; ^3^ Department of Bio-Medical Sciences, Philadelphia College of Osteopathic Medicine, Philadelphia, PA, United States

**Keywords:** BRCA2, PALB2, prostate cancer, platinum-based chemotherapy, radiotherapy, case report

## Abstract

**Background:**

Mutation-caused loss-of-function of factors involved in DNA damage response (DDR) is responsible for the development and progression of ~20% of prostate cancer (PCa). Some mutations can be used in cancer risk assessment and informed treatment decisions.

**Methods:**

Target capture-based deep sequencing of 11 genes was conducted with total DNA purified from the proband’s peripheral blood. Sanger sequencing was conducted to screen potential germline mutations in the proband’s family members. Targeted sequencing of a panel of 1,021 genes was done with DNA purified from the tumor tissue.

**Results:**

Two previously unreported germline mutations in the DDR pathway, *BRCA2* (c.8474_8487delCATACCCTATACAG, p.A2825Vfs*15) and *PALB2* (c.472delC, p.Q158Rfs*19) were identified in a patient with metastatic PCa. A specific therapeutic regimen including androgen deprivation therapy, locally radical radiotherapy, and systemic platinum chemotherapy worked well against his cancer. In addition, the metastatic ovarian cancer in the proband’s half-sister harboring the same *BRCA2* germline mutation also responded well to platinum chemotherapy.

**Conclusions:**

The newly identified germline mutations in DDR plays important role in PCa development. Since specific regimen worked well against this cancer, screening of DDR mutation could provide better management for patients with these mutation-mediated PCa.

## Introduction

Prostate cancer (PCa) is the most prevalent cancer in men and the second leading cause of cancer-related death worldwide. It has been estimated that in the United States there will be 191,930 new PCa diagnoses and 33,330 PCa-related deaths in 2020 (1). Compared with the general population, first-degree relatives of men with PCa have approximately twice the risk of developing PCa (2) and genetic mutations are responsible for ~42% of this disease (3). Genome-wide association studies have identified more than 100 common variants that account for approximately 33% of familial PCa risk (4). Multiple lines of evidence suggest that mutation in genes involved in DNA damage response (DDR) plays rather important role in cancer development and progression (5–7).

It has been estimated that the mutation of genes in DDR is responsible for at least 19% of localized PCa (6) and 23% of metastatic castration-resistant PCa (7). Proteins encoded by *BRCA2*, *BRCA1*, *ATM*, *CHEK2*, *PALB2*, and mismatch repair (MMR) genes including *MSH2* and *MSH6* play important role in DDR (7). Mutations in some of these genes have already been used for risk assessment and treatment decision-making (8). For example, germline mutation of *BRCA* usually confers a more aggressive PCa with higher Gleason scores (9), a higher probability of nodal involvement, distant metastasis, and shorter overall survival (10). More importantly, patients with advanced PCa harboring DDR gene mutations generally respond well to poly (ADP) ribose polymerase (PARP) inhibitors and platinum-based chemotherapy (11, 12). The U.S. Food and Drug Administration (FDA) recently approved two poly-ADP ribose polymerase (PARP) inhibitors, olaparib (11, 13), and rucaparib (14), as treatments for patients with metastatic castration resistant adenocarcinoma of the prostate harboring deleterious or suspected deleterious germline or somatic HRR gene-mutations. Therefore, the stratification of PCa patients with mutations in DDR pathway may lead to more informed therapies.

We here report a patient with metastatic PCa carrying previously unreported germline mutations in *BRCA2* and *PALB2*, two important players in DDR. More importantly, this patient responded well to a specific therapeutic regimen including androgen deprivation therapy, locally radical radiotherapy, and systemic platinum chemotherapy. In addition, his half-sister carrying the same *BRCA2* mutation with metastatic ovarian cancer responded equally well to platinum chemotherapy.

## Materials and Methods

### Patients

All procedures involving human participants were carried out in accordance with ethical standards of the institutional research committee at the Army Medical University in Chongqing, China and with the 1964 Helsinki Declaration and its later amendments or comparable ethical standards. All patients provided written, informed consent for review of their medical record and sequence of their primary and/or metastatic PCa tissue. The research was conducted with Army Medical University IRB approval.

### Identification of the Mutations

Total DNA was extracted from either the patient’s leukocytes, primary PCa, or metastatic lung cancer tissues using the QIAamp DNA Micro Kit (Qiagen, Hilden, Germany) according to the manufacturer’s instructions. Target capture-based deep sequencing (BGI Health, China) was conducted to screen potential mutations in a panel of 11 genes (*ATM*, *BRCA1*, *BRCA2*, *MLH1*, *MLH3*, *MSH2*, *MSH3*, *MSH6*, *PALB2*, *PMS1*, and *PMS2*) using DNA purified from the proband’s peripheral blood. Sanger sequencing was conducted to screen potential germline mutations in the proband’s family members. Targeted sequencing of a panel of 1021 genes (**Supplementary Table S1**) was done with DNA purified from the tumor tissue by Geneplus-Beijing Institute (Beijing, China).

## Results

### Case Presentation

**Figure 1:**
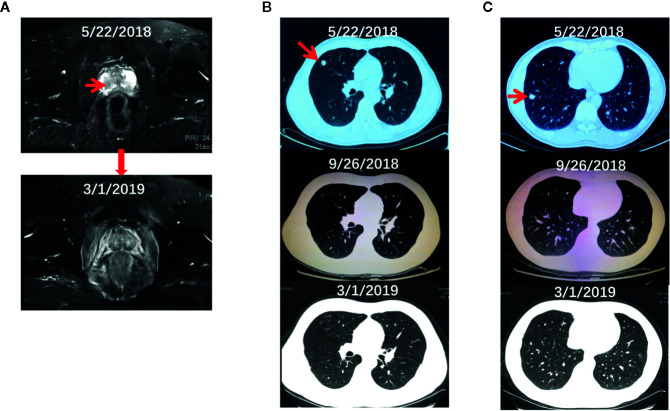
The radiographs of the proband before and during the treatment. **(A–C)** The pelvic MRI **(A)** and chest CT **(B, C)** scan of the proband before and after the systematic treatment.

The proband is a 48-year-old Chinese male who presented to our department on July 4, 2018 after having hematuria for 2 months. Digital rectal examination found the right lobe of his prostate is hardened with irregularities. Laboratory tests showed a relatively normal level of total serum PSA (prostate-specific antigen, 3.03 ng/ml). Pelvic magnetic resonance imaging (MRI) showed a lesion (3.1 × 4.3 cm) in the peripheral zone of the right lobe of his prostate with a low-intensity signal on T2 weighted imaging (**Figures 1A**, upper panel****). Ultrasound-guided transrectal prostate biopsies were conducted and pathological examination showed prostate adenocarcinoma in 4 of the 14 cored biopsies with an average Gleason score 4 + 4. Computed tomography (CT) chest scan also revealed two small lesions in his right lung (**Figures 1B, C**, upper panel****). Whole body positron emission tomography (PET)-CT scan found hypermetabolic lesions in both his prostate and right side of lung, but not in the bone or other organs. Immunohistochemistry of the lesions from the right lung showed positive staining of PSA and negative staining of CDX-2 and TTF-1 (data not shown). Therefore, his diagnose was a primary PCa with lung metastasis (T2cNxM1c).

### Identification of Germline and Somatic Mutations

**Figure 2:**
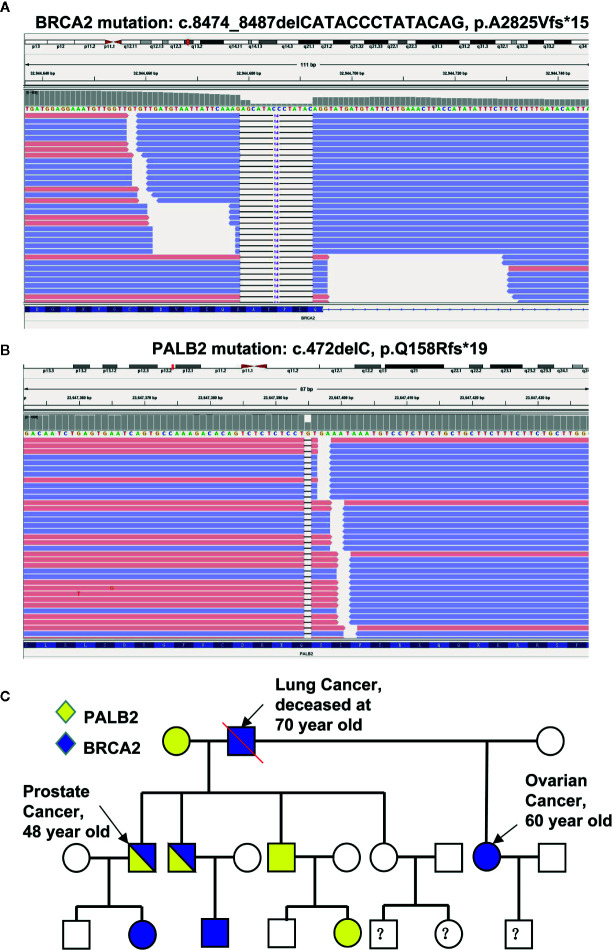
Identification of mutations in the proband and his family members. **(A, B)** Sequencing reads of *BRCA2* **(A)** and *PALB2* **(B)** are shown by the Integrative Genomic Viewer. **(C)** The pedigree of mutations.

Given that (i) the relative early-onset and high aggressiveness of cancer, (ii) his father died of lung cancer at the age of 70, and (iii) his half-sister suffered from metastatic ovarian cancer with severe ascites in her early 60s, we decided to screen potential germline mutations. To do so, DNA extracted from the patient’s leukocytes was used for sequencing 11 genes involved in the DDR pathway including *ATM*, *BRCA1*, *BRCA2*, *MLH1*, *MLH3*, *MSH2*, *MSH3*, *MSH6*, *PALB2*, *PMS1*, and *PMS2*. Two previously unreported germline mutations of *BRCA2* (c.8474_8487delCATACCCTATACAG, p.A2825Vfs*15, **Figure 2A**) and *PALB2* (c.472delC, p.Q158Rfs*19, **Figure 2B**) were identified. These deletions result in the expression of truncated *BRCA2* and *PALB2*. Next, leukocyte DNA was isolated from the other 10 immediate family members of the proband and used for Sanger sequencing of the *BRCA2* and *PALB2* genes. The characteristics of the family members and their genetic mutations were summarized in **Table 1**. Based on the pedigree (**Figure 2C**), we postulated that the proband inherited his mutant *BRCA2* allele from his father and the mutant *PALB2* from his mother and therefore the proband carries a heterozygous mutation of both *BRCA2* and *PALB2*. We then decided to screen any potential somatic mutations. Total DNA extracted from both his prostate and lung cancer tissues was used to sequence a panel of 1021 genes highly involved in PCa. In addition to the germline mutant *BRCA2* and *PALB2*, 10 and 9 additional somatic mutations were identified in his prostate and lung lesions, respectively (**Supplementary Table S2**). Of note, the six mutations with the highest frequency (>10%) including *PAG1* (c.702A>T, p.K234N), *KDM5C* (c.1869G>C, p.L623F), *CDH11* (c.1048G>A, p.A350T), *AFF2* (c.124_141delGATCTCTTTTCTTCAGGC, p.D42_G47del), *FOXA1* (c.753_764delCAACATGTTCGA, p.M253_N256del), and *HCLS1* (c.2_7dupTGTGGA, p.M1_W2dup) were identical in both the lung and the prostate lesions. These data strongly suggest that the lesions in his right lung were metastasized from his PCa.

**Table 1:** The characteristics of the patient and his family members.

Individuals	Gender	Age (years old)	Carrying mutations	With Cancers
The proband	Male	48	BRCA2 and PALB2	Prostate cancer
His father	Male	Died at 70	BRCA2	Lung cancer
His mother	Female	80	PALB2	Not yet
His son	Male	16	No	No
His daughter	Female	19	BRCA2	Not yet
The proband’s half-sister	Female	60	BRCA2	Ovarian cancer
Her son	Male	36	Unknown	Unknown
The proband’s older brother	Male	54	BRCA2 and PALB2	Unknown
His son	Male	31	BRCA2	Not yet
The proband’s younger brother	Male	45	PALB2	Not yet
His son	Male	24	No	No
His daughter	Female	9	PALB2	Not yet
The proband’s sister	Female	51	No	No
Her son	Male	30	Predicted No	Predicted No
Her daughter	Female	22	Predicted No	Predicted No

### Treatments and Responses

**Figure 3:**
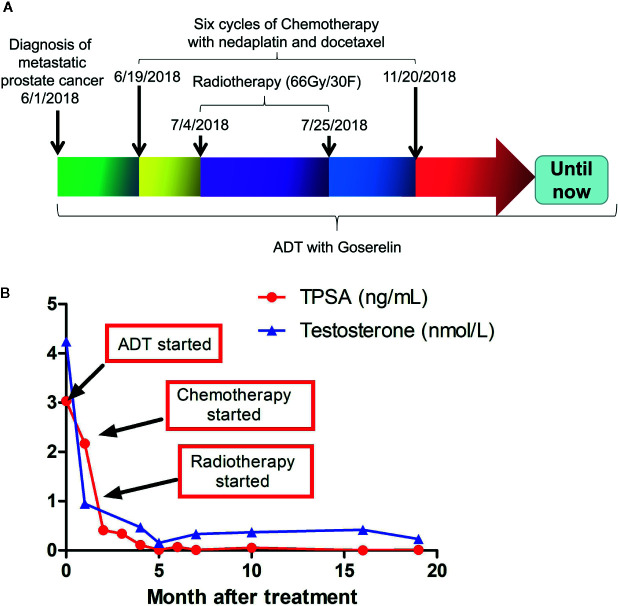
Treatment regimens and laboratory test results during and after the treatment. **(A)** therapeutic schedules for the proband. **(B)** Serum levels of TPSAand testosterone in the proband during and after treatment. TPSA, total prostate-specific antigen.

The treatment regimen for the proband is shown in **Figure 3A**. Based on the recommendation from the NCCN guideline for M1 castration-naive PCa (15), primary ADT (androgen deprivation therapy) with Goserelin (10.8mg sc 1/3months) started on the date of diagnosis and continued for the rest of the treatment. Between June 29, 2018, and November 20, 2018, six cycles of chemotherapy with the combination of nedaplatin (130 mg, formula: 75 mg/m^2^ × body surface area) and docetaxel (130 mg, formula: 75 mg/m^2^ × body surface area) were administrated. His body surface area was about 1.75 m^2^ based on the formula 0.0061 × height (167 cm) + 0.0124 × weight (60 kg)-0.0099. In addition, EBRT (External Beam Radiation Therapy; 66 Gy/30 F, 2.2 Gy/F) was applied to the local primary PCa between June 4^th^ and June 25^th^. Only mild adverse events such as slightly reduced leucocyte count were seen during the whole regimen. Workup to evaluate the treatment included the levels of serum PSA and testosterone (**Supplementary Table S3**), bone imaging, Chest CT and pelvic MRI without contrast. **Figure 3B** showed that the level of serum testosterone decreased to castrated level 4 months after the beginning of ADT. The levels of serum TPSA became and remained nearly undetectable 5 months after the start of ADT. The concentration of TPSA was 0.01 ng/ml 15 months after the start of the treatment. Pelvic MRI (March 2, 2019) revealed that the prostate volume shrank markedly and the tumor was barely detectable (**Figure 1A**, bottom panel) 10 months after the start of ADT. Chest CT scans conducted on September 26, 2018 (**Figures 1B, C**, middle panel) and March 1, 2019 (**Figures 1B, C**, bottom panel) and PET-CT examination on October 3, 2019 (data not shown) found that the lesions in his right lung disappeared completely. ADT has been continued without any sign of disease progression up to the preparation of this report. In addition and according to the recommendation of the NCCN guideline (16), we have also treated his half-sister’s metastatic ovarian cancer with platinum-based chemotherapy. After six cycles of chemotherapy, her general vital signs including appetite and physical energy improved greatly. More importantly, the ascites disappeared, and the tumors in her ovaries and abdominal cavity did not progress as of the preparation of this report.

## Discussion

We report in this study a PCa patient carries previously unreported germline mutation of *BRCA2* and *PALB2* and the same *BRCA2* mutation was found in his half-sister who suffered from metastatic ovarian cancer. In addition, the PCa in the proband has also metastasized to his right lung. The DNA sequencing of the proband’s family members showed that the proband’s brother carries the same *BRCA2* and *PALB2* mutations. In addition, six individuals carry either the mutant *BRCA2* (three individuals) or *PALB2* (three individuals) in his first- and second-degree relatives (**Figure 2C**). The proband underwent ADT and platinum-based chemotherapy as well as local radiation for his PCa, and his half-sister with metastatic ovarian cancer was treated with standard platinum-based chemotherapy. Both patients responded extremely well to the regimens, although the follow-up duration is relatively short. Particularly, the proband was found with an undetectable level of PSA, barely detectable PCa, and totally disappeared lung metastases after the systemic treatments.

Germline mutation in genes involved in Lynch syndrome (*MSH2*, *MSH6*, and *MLH1*) and those in homologous recombination (*BRCA1*, *BRCA2*, *ATM*, *PALB2*, and *CHEK2*) increase not only the incidence but also aggressiveness of multiple cancer types including prostate, breast, and ovarian cancer. Cancers with these mutations usually also have poorer outcomes (17, 18). Consistent with the findings that mutations of genes in the DDR pathway such as *BRCA2*, *PALB2*, and *ATM* play important roles in PCa (19), we report a PCa patient with *BRCA2* and *PALB2* double mutations. Germline *BRCA2* mutations were found in 5.35% of PCa patients from Caucasian (4) and 6.3% from Chinese population (20). BRCA2 is a protein comprised of 3418 amino acid residues and functions as a scaffold to form a multiprotein complex with Rad51, BRCA1. This complex acts as a caretaker of genome integrity by enabling HR (homologous recombination)-based double-strand DNA break repair and intra-S phase DNA damage checkpoint control. The germline mutant *BRCA2* (c.8474_8487delCATACCCTATACAG, p.A2825Vfs*15) identified in this research encodes a truncated protein with 2840 amino acid and lack the 578 residuals at its C-terminus. Since the function of BRCA2 is severely affected when the 110 residuals at its C-terminus are lost (21), the truncated *BRCA2* identified in the current report likely encode a loss-of-function BRCA2. Previous studies have demonstrated that loss of heterozygosity (LOH) occurred in most of *BRCA* carriers, including 100% ovarian cancer with germline *BRCA1* mutation (22) and 67% PCa with germline *BRCA2* mutation (23). Therefore, we evaluated the LOH of *BRCA2* and *PALB2* in our case by using allele frequency comparisons (22). Based on the HE staining of the biopsy specimens, we estimated the percentage of tumor cells in the biopsy specimens and found that ~80% of the lung biopsy tissue is composed of tumor cells. However, only about 50% of the prostate biopsy sample is tumor cells. We have also noticed that the variant allele frequencies (VAF) of the mutation *PAG1* (c.702A>T) in the lung and prostate biopsy samples were 73.8% and 48.2% (**Supplementary Table S2**), respectively, proportional to the abovementioned tumor cell percentage, which indicating that the mutation was homozygous. Since the VAF of *BRCA2* in lung and prostate biopsy tissues are very close to that of *PAG1* in these tissues, we inclined to conclude that *BRCA2* mutation in both lung and PCa cells is homozygous resulted from loss-of-heterozygosity in both cancers. On the other hand, since the VAF of *PALB2* is about 50% of *PAG1’s* VAF in both tissues, we conclude that loss-of-heterozygosity did not happen to *PALB2* gene in these cancer cells. However, due to lacking any experimental evidence, we were unable to unequivocally conclude that either or both of them are driver genes in PCa development. Nevertheless, based on their roles in DNA damage repair especially additional 10 and 9 somatic mutations were found in prostate and lung lesions, respectively (**Supplementary Table S2**), we propose that these mutations play important roles in PCa initiation/progression.

Multiple lines of evidence indicate that PCa with germline *BRCA2* mutations is more aggressive than those with sporadic mutations (17, 18). In addition, double mutations of members in the DDR pathway not only confer an early onset but also a more aggressive phenotype of the tumors (24, 25). The proband also carries a germline mutation in *PALB2* (c.472delC, p.Q158Rfs*19), another member of the DDR pathway. It has reported that the mutant *PALB2* (c.1592delT) encodes a loss-of-function PALB2 (26), which is unable to mediate BRCA2 nuclear localization/accumulation and led to HR/DSBR (double strand break repair) deficiency (27). Since the truncated peptide expressed in the proband is even shorter than the previously reported PALB2 expressed from the *PALB2* (c.1592delT), it is conceivable that the DDR pathway in the proband with co-mutation of *BRCA2* and *PALB2* would be affected more severely than those with either one alone. Since the PCa of the proband is encapsulated (T2c) and the level of TPSA (3.03 ng/ml) is not elevated, the tumor in his prostate is likely to be still at its early stage. However, given that (i) his lung cancer is positive of PSA and negative of both CDX-2 and TTF-1, (ii) the extremely high similarity between the mutations found in the lung and prostate lesions with the top six mutations identical, he was diagnosed as a primary PCa with lung metastases, an extremely rare case. Since the PCa has already metastasized to the lung but not the lymph node nor the bone although these organs are the most common metastatic sites of PCa (28), we speculated that PCa with germline co-mutation of *BRCA2* and *PALB2* might be more aggressive. Therefore, his brother carrying the same double mutations could also have a higher risk of developing an advanced PCa.

Visceral metastases are commonly associated with advanced castration-resistant PCa, prolonged treatment, and neuroendocrine PCa (29, 30). Primary PCa with solitary lung metastasis has rarely been reported with a low incidence of 0.2% (31) and we report a PCa patient with solitary distant metastases to the lung who was found with germline co-mutation of *BRCA2* and *PALB2*. A recent study (32) has shown that mHSPC (metastatic Hormone-Sensitive Prostate Cancer) with lung-only metastases might be an unique molecular and clinical subgroup with significant enrichment for MMR (25%) and HDR (25%) mutations. Of note, two patients with solitary lung metastases were found with germline mutations of *BRCA2*. Moreover, that study demonstrated that these patients showed favorable clinical outcomes to first-line ADT treatments. Although ADT is the gold standard for patients with metastatic PCa, a universally accepted regimen for these kinds of patients is lacking. Based on the report that (i) mCRPC in patients with biallelic mutant *BRCA2* responded well to platinum chemotherapy (33) and (ii) localized PCa with mutant BRCA can be treated with radiotherapy effectively (34, 35), we carefully crafted a regimen tailored to the proband (**Figure 3A**) and the patient responded to the treatment well (**Figure 3B**). In addition, his half-sister with metastatic ovarian cancer and the same germline mutation of *BRCA2* also responded to platinum-based chemotherapies particularly well. Since cells with HR-deficiencies cannot repair DNA-damage efficiently, the cancer cells in the patients reported here would be more prone to chemo- and/or radiotherapy-mediated cancer cell apoptosis (36–38). We do acknowledge that an 18-month follow-up for PCa is too short to make any solid conclusion for the long-term effect of this regimen. The finding reported here are the first of its kind. We believe that longer follow-up and further research on a larger cohort of patients with these mutations will undoubtedly provide a more solid conclusion for the long-term effect of our therapeutic regimen. In addition, we could not simply attribute the current therapeutic effect to platinum chemotherapy, because ADT and docetaxel might also result in favorable outcomes during such short follow-up (32). Even so, the therapeutic effect of platinum chemotherapy on this patient should be highlighted. Just as Mark M. Pomerantz et al. showed that carboplatin-based chemotherapy could render better prognosis in patients with *BRCA2* germline mutations than those without (39), although *BRCA2* mutations are associated with more aggressive PCa. Given that PARP inhibitor olaparib and rucaparib can improve progression-free survival for mCRPC patients with mutations in DNA-repair genes (11), olaparib and rucaparib could be an alternative treatment for patients with *BRCA2* and/or *PALB2* mutations. TOPARP-A (11) and TOPARP-B (13) trails have revealed that olaparib, an orally bioavailable inhibitor of the catalytic activity of PARP1 and PARP2, has antitumor activity against metastatic castration-resistant PCa with specific DDR gene aberrations. More recently, the phase II TRITON2 study has found that the PARP inhibitor rucaparib has antitumor activity in mCRPC patients with a deleterious *BRCA* alteration (14) and specific non-*BRCA* DDR gene (e.g., *PALB2*) alteration (40). In addition, additional clinical trials are in progress for talazoparib, velinarib and niraparib (41).

In summary, we identified two previously unreported germline mutations in the DNA double-strand repair pathway, *BRCA2* and *PALB2* in a PCa patient with solitary lung metastasis but without bone lesion (T2cNxM1c). Since there is no consensus treatment for these patients, we designed a therapeutic regimen including androgen deprivation therapy, systemic platinum chemotherapy and locally radical radiotherapy specifically tailored to his prostate tumor and the patient responded well. These findings support the guidelines and consensus statements from international clinical organizations (42, 43) that recommend DDR mutation screening for the management of particular PCa patients. More importantly, these newly identified mutations in DDR are associated with PCa and can serve as the base for designing personalized treatment.

## Data Availability Statement

The original contributions presented in the study are included in the article/**Supplementary Material**. Further inquiries can be directed to the corresponding authors.

## Ethics Statement

The studies involving human participants were reviewed and approved by Army Medical University IRB. The patients/participants provided their written informed consent to participate in this study. Written informed consent was obtained from the individual(s) for the publication of any potentially identifiable images or data included in this article.

## Author Contributions

Conception/design: JJ and QL. Provision of study material or patients: TT and L-aW. Collection and/or assembly of data: TT, L-aW, PW, DT, GL, and GY. Data analysis and interpretation: JZ, YZ, ND, and JJ. Manuscript writing and revising: TT, L-aW, QL, KG, DZ, and JJ. All authors contributed to the article and approved the submitted version.

## Funding

This work was supported by University Research Project of Army Medical University (2017XYY07, JJ).

## Conflict of Interest

The authors declare that the research was conducted in the absence of any commercial or financial relationships that could be construed as a potential conflict of interest.

## Abbreviations

DDR, DNA damage response; PCa, Prostate cancer; MMR, mismatch repair; PARP, poly (ADP) ribose polymerase; PSA, prostate-specific antigen; MRI, magnetic resonance imaging; CT, Computed tomography; PET, Positron emission tomography; ADT, androgen deprivation therapy; EBRT, External Beam Radiation Therapy; HR, homologous recombination; DSBR, double strand break repair.
