# Supplementary material for: Case Report: Co-Existence of BRCA2 and PALB2 Germline Mutations in Familial Prostate Cancer With Solitary Lung Metastasis
Source: Front Oncol. 2020 Oct 26;10:564694. doi: 10.3389/fonc.2020.564694 (PMC7649358; doi:10.3389/fonc.2020.564694)
Supplement: Supplementary file 2 [file Table_2.docx]

**Supplementary Table S2.** The somatic mutations in the prostate and lung tissues of the patient.

| Genes | Mutations (Frequency) | |
| --- | --- | --- |
|  | Prostate tissue | Lung tissue |
| PAG1 | c.702A>T, p.K234N (48.2%) | c.702A>T, p.K234N (73.8%) |
| KDM5C | c.1869G>C, p.L623F (38.0%) | c.1869G>C, p.L623F (86.0%) |
| CDH11 | c.1048G>A, p.A350T (21.6%) | c.1048G>A, p.A350T (38.6%) |
| AFF2 | c.124_141delGATCTCTTTTCTTCAGGC, p.D42_G47del (19.9%) | c.124_141delGATCTCTTTTCTTCAGGC, p.D42_G47del (45.4%) |
| FOXA1 | c.753_764delCAACATGTTCGA, p.M253_N256del (14.9%) | c.753_764delCAACATGTTCGA, p.M253_N256del (33.2%) |
| HCLS1 | c.2_7dupTGTGGA, p.M1_W2dup (10.9%) | c.2_7dupTGTGGA, p.M1_W2dup (24.5%) |
| MITF | c.694A>T, p.S232C (6.0%) |  |
| LRP5 | c.4249G>T, p.G1417W (2.3%) |  |
| MLL | c.11219C>T, p.A3740V (2.3%) |  |
| EXT1 | c.1973A>C, p.N658T (1.5%) |  |
| ASXL1 |  | c.776T>G, p.L259R (2.5%) |
| SRC |  | c.1310C>G, p.A437G (1.0%) |
| POLE |  | c.3043G>T, p.D1015Y (1.0%) |
