# Supplementary material for: Case Report: Co-Existence of BRCA2 and PALB2 Germline Mutations in Familial Prostate Cancer With Solitary Lung Metastasis
Source: Front Oncol. 2020 Oct 26;10:564694. doi: 10.3389/fonc.2020.564694 (PMC7649358; doi:10.3389/fonc.2020.564694)
Supplement: Supplementary file 3 [file Table_3.docx]

| Time | TPSA (ng/ml) | FPSA (ng/ml) | Testosterone (nmol/L) |
| --- | --- | --- | --- |
| 2018/5/21 | 3.03 | 0.70 | 4.24 |
| 2018/6/20 | 2.17 | 0.64 | 0.95 |
| 2018/7/15 | 0.41 | 0.07 |  |
| 2018/8/7 | 0.34 | 0.05 |  |
| 2018/9/26 | 0.11 | 0.03 | 0.47 |
| 2018/10/24 | 0.01 | 0.01 | 0.15 |
| 2018/11/21 | 0.07 | 0.04 |  |
| 2018/12/18 | 0.01 | 0.00 | 0.33 |
| 2019/3/2 | 0.05 | 0.04 | 0.37 |
| 2019/9/18 | 0.00 | 0.00 | 0.42 |
| 2020/1/1 | 0.01 | 0.08 | 0.23 |

**Supplementary Table S3. The levels of TPSA, FPSA and testosterone of the patient during the treatment.**
